# Liver–lung interactions in acute respiratory distress syndrome

**DOI:** 10.1186/s40635-020-00337-9

**Published:** 2020-12-18

**Authors:** Raquel Herrero, Gema Sánchez, Iris Asensio, Eva López, Antonio Ferruelo, Javier Vaquero, Laura Moreno, Alba de Lorenzo, Rafael Bañares, José A. Lorente

**Affiliations:** 1 Department of Critical Care Medicine, Hospital Universitario de Getafe, Madrid, Spain; 2 CIBER de Enfermedades Respiratorias, Instituto de Investigación Carlos III, Madrid, Spain; 3 Fundación de Investigación Biomédica del Hospital Universitario de Getafe, Madrid, Spain; 4 Laboratory of Biochemistry, Hospital Universitario de Getafe, Madrid, Spain; 5 Servicio de Aparato Digestivo. HGU Gregorio Marañón, Instituto de Investigación Sanitaria Gregorio Marañón (IiSGM), Madrid, Spain; 6 CIBER de Enfermedades Hepáticas y Digestivas, Instituto de Investigación Carlos III, Madrid, Spain; 7 Department of Pharmacology, School of Medicine, Universidad Complutense de Madrid, Madrid, Spain; 8 Universidad Europea de Madrid, Madrid, Spain

**Keywords:** Liver–lung interaction, Acute respiratory distress syndrome, Liver dysfunction, Mechanisms, Immunomodulation, Acute-phase response, Critical illness

## Abstract

Patients with liver diseases are at high risk for the development of acute respiratory distress syndrome (ARDS). The liver is an important organ that regulates a complex network of mediators and modulates organ interactions during inflammatory disorders. Liver function is increasingly recognized as a critical determinant of the pathogenesis and resolution of ARDS, significantly influencing the prognosis of these patients. The liver plays a central role in the synthesis of proteins, metabolism of toxins and drugs, and in the modulation of immunity and host defense. However, the tools for assessing liver function are limited in the clinical setting, and patients with liver diseases are frequently excluded from clinical studies of ARDS. Therefore, the mechanisms by which the liver participates in the pathogenesis of acute lung injury are not totally understood. Several functions of the liver, including endotoxin and bacterial clearance, release and clearance of pro-inflammatory cytokines and eicosanoids, and synthesis of acute-phase proteins can modulate lung injury in the setting of sepsis and other severe inflammatory diseases. In this review, we summarized clinical and experimental support for the notion that the liver critically regulates systemic and pulmonary responses following inflammatory insults. Although promoting inflammation can be detrimental in the context of acute lung injury, the liver response to an inflammatory insult is also pro-defense and pro-survival. A better understanding of the liver–lung axis will provide valuable insights into new diagnostic targets and therapeutic strategies for clinical intervention in patients with or at risk for ARDS.

## Background

**Fig. 1:**
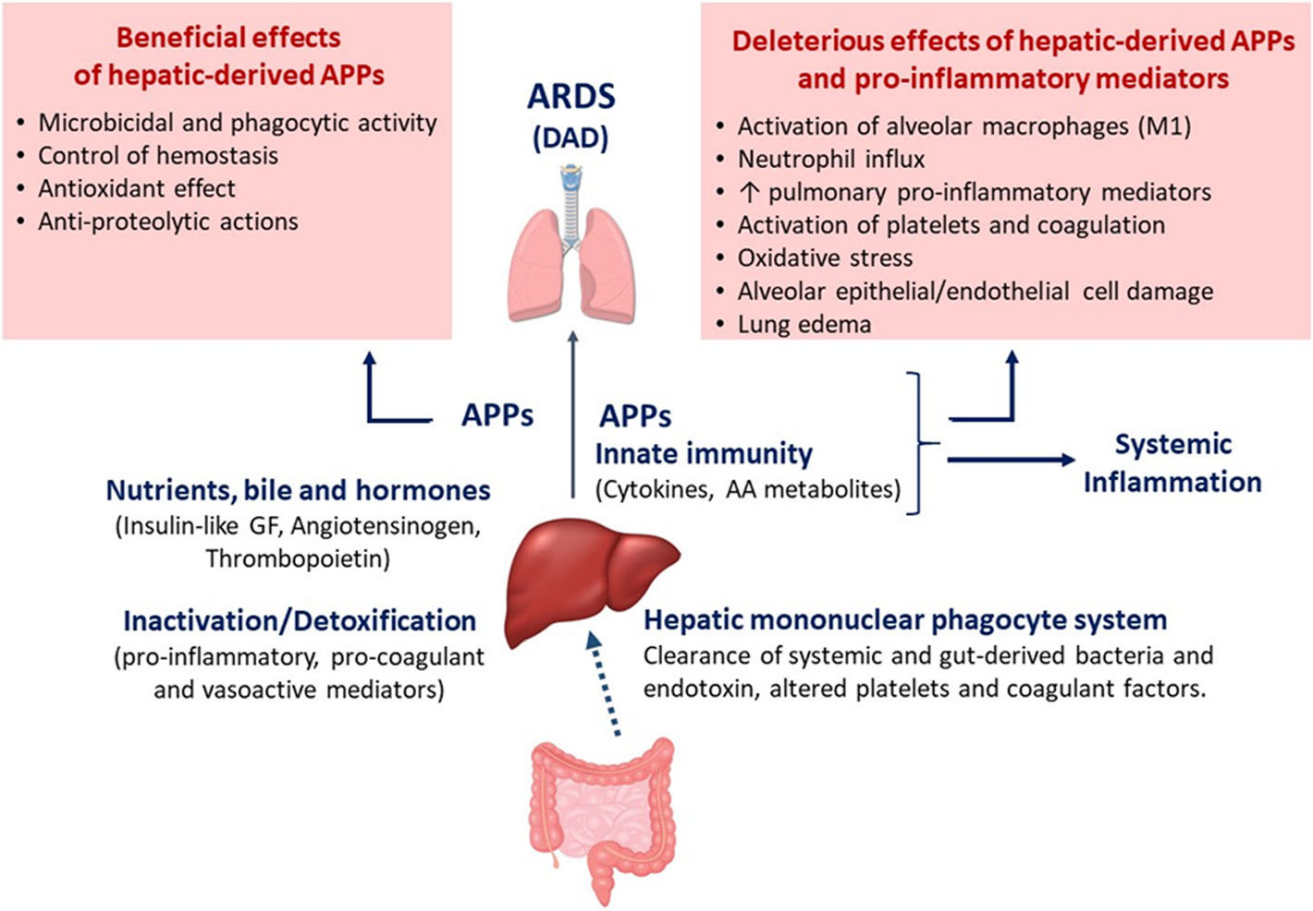
Role of the gut–liver–lung axis in acute respiratory distress syndrome. There are several physiological mechanisms promoted by the liver that contribute to the development, progression, and resolution of the acute respiratory distress syndrome. *ARDS* acute respiratory distress syndrome, *DAD* diffuse alveolar damage, *APPs* acute-phase proteins, *AA* arachidonic acid, *GF* growth factor

Acute respiratory distress syndrome (ARDS) is a severe respiratory failure, due to non-cardiogenic pulmonary edema [1, 2], associated with a hospital mortality between 35% and 46% [1, 3, 4]. The pathology of ARDS involves diffuse alveolar damage (DAD), which comprises severe alveolar epithelial cell damage, neutrophil infiltration, activation of alveolar macrophages, production of cytokines and chemokines, plasma extravasation, procoagulant activity with fibrin deposition, hyaline membrane formation, myofibroblast proliferation, and fibrosis in the intra-alveolar spaces [2, 4]. Formation of protein-rich edema in the airspaces due to the disruption of the alveolar–capillary membrane is one of the main factors that contributes to the severe impairment of blood and tissue oxygenation early in the evolution of DAD [2, 4]. The DAD occurs not only in response to a direct injury to the lung (e.g., pneumonia), but it may also represent a pulmonary manifestation of diverse systemic immunoregulatory disorders, such as sepsis [4]. The pathogenesis of ARDS, therefore, is linked to changes in local and systemic host defense and immune responses [5], in which the liver plays an important role (Fig. 1).

The liver has unique anatomic, cellular, and physiological characteristics that enable the clearance of circulating microbial products, tissue debris, altered platelets, products of intravascular coagulation, and different bioactive molecules (Fig. 1) [6–10]. Also, the liver has a key role in the synthesis of proteins, metabolism of toxins and drugs, and in the modulation of systemic inflammatory responses and host defense (Fig. 1). It is becoming more evident that normal liver function exerts lung protection and is necessary for recovery from lung damage [11, 12]. In this line, it has been observed that established ARDS during acute liver allograft rejection is resolved within hours of hepatic re-transplantation [13]. On the other hand, experimental studies suggest that the presence of the liver is also absolutely necessary for inducing lung injury in rats [14]. These apparently paradoxical observations highlight the relevant crosstalk between lung and liver in ARDS.

Despite the well-recognized liver–lung interaction in the pathogenesis of ARDS, its underlying mechanisms and its effects on the outcome of these patients have been barely studied due to several reasons. First, patients with liver diseases are frequently excluded from studies of ARDS. In addition, liver function is not precisely reflected by the standard liver function tests in the clinical setting, and the liver is not as accessible as other organs such as the lung, making liver dysfunction not as evident as dysfunctions of other organs. Finally, its clinical consequences are also heterogeneous in critically ill patients [15]. The present work reviews the important role of the liver on the development and resolution of ARDS and aims to provide an integrated view of the underlying mechanisms that support the liver–lung interaction in critically ill patients.

## The reciprocal impacts of lung and liver dysfunctions

Following hepatocellular damage, the liver may reduce its clearance function, increase the synthesis of deleterious substances, and dysregulate immune responses, leading to systemic complications such as coagulopathy, elevated risk of infection, hypoglycaemia, exacerbated inflammatory responses, encephalopathy, and damage of other extrahepatic organs, including lung injury [16–22]. In critically ill patients, hepatic dysfunction is recognized as a relevant clinical condition that influences the development, severity, and progression of ARDS [5, 11, 19, 23–27]. In ARDS patients, liver dysfunction is a major determinant of mortality [24–26]. It is well known that cirrhosis and other chronic liver diseases make the patients more susceptible for developing ARDS, which adversely affects patient outcomes [24–26, 28]. A growing body of evidence suggests that liver damage activates and enhances inflammation in the pulmonary intravascular compartment and lower respiratory tract, leading to important changes in the structure and/or functions of the lung [29, 30]. Although all these observations indicate that liver function is an important factor for the development and resolution of ARDS, there is also evidence that such interorgan communication is bidirectional. Thus, acute lung injury is known to impair hepatic function and to aggravate liver diseases by mechanisms involving hypoxemia, activation of systemic inflammatory responses, and cardiovascular changes [24, 31, 32].

## Liver dysfunction is common in critical care patients

The frequency of liver damage in critical illness has considerably increased over the last decades [23, 33–35], reaching up to 20% of ICU patients in some series and elevating their morbi-mortality [33, 34, 36]. In critically ill patients, liver dysfunction usually occurs after inflammatory insults such as sepsis and trauma [15, 23, 33, 37], and the underlying interactive mechanisms are complex. The mechanisms of liver dysfunction in critically ill patients implicate microbial products, the paracrine action of cytokines and other inflammatory mediators, hypoxemia, oxidative stress, toxic compounds, hypoperfusion, passive congestion, and effect of nutrition support, among others [34, 38–40].

Liver dysfunction can be manifested by plasma elevation of liver enzymes (aspartate aminotransferase (AST), alanine aminotransferase (ALT), alkaline phosphatase (ALP), γ-glutamyl transpeptidase) and bilirubin, decreased plasma levels of albumin and coagulation factors, and/or increased international normalized ratio (INR) [33, 36]. The clearance rate of indocyanine green has been used as a dynamic test to assess the functional capacity of the liver. Although the indocyanine green test has shown to reflect better the excretory and/or microvascular dysfunction of the liver, its clinical use has certain limitations [41]. Increased plasma levels of bilirubin are associated with high mortality in critically ill patients [39, 42]. Furthermore, hyperbilirubinemia has been proposed as a biomarker of ARDS and found to be an independent factor of mortality in patients with ARDS [26, 35, 39, 43]. Unfortunately, neither bilirubinemia nor other hepatic parameters routinely measured in the clinical setting have the sensitivity and specificity required for an early identification of hepatic injury in critically ill patients [23, 33, 36, 40].

## Mechanisms of liver–lung interactions in ARDS

The mechanisms by which the liver modulates lung injury involve interrelated elements of systemic and pulmonary host defense, inflammatory responses, and metabolism and include the following (see Fig. 1).

### Clearance by the hepatic mononuclear phagocyte system of systemic endotoxemia, bacteremia, vasoactive by-products, and procoagulant factors

The mononuclear phagocyte system located in the liver, spleen, lung, and bone marrow constitutes the major mechanism to uptake and detoxify bacteria, fungi, viruses, and dying cells, limiting the magnitude and duration of infections [10, 44, 45]. Although these mononuclear phagocytic cells can exert this function in all these locations, their major mass is in the hepatic sinusoids [10, 46]. The hepatic sinusoid is a unique vascular structure with highly specialized endothelial cells (liver sinusoidal endothelial cells) and liver macrophages (Kupffer cells) that reside within the lumen. The cells of the hepatic sinusoid are constantly exposed to gut-derived bacteria, microbial debris, and bacterial endotoxins. Kupffer cells, which line the extensive sinusoidal network, constitute nearly 80–90% of the tissue macrophages present in the body and exert an important role in host defense through phagocytosis and a multitude of secretory functions [46]. The hepatic mononuclear phagocyte system acts as a first line of defense in clearing bacteria and their products. Besides uptake of microbial pathogens and products, Kupffer cells also protect the lung and other extrahepatic organs by removing altered platelets and intravascular coagulation products (Fig. 1) [8–10, 46].

Dysfunction of the reticuloendothelial system of the liver allows bacterial and microbial products, including the so-called pathogen-associated molecular patterns (PAMs) [47], to reach the lung and the systemic circulation, where they activate pulmonary and systemic inflammatory responses (Fig. 1) [29, 47–49]. Indeed, increased plasma levels of endotoxin, probably of intestinal origin, along with increased levels of some cytokines have been found in the blood of patients with acute and chronic liver diseases [50–54].

**Fig. 2:**
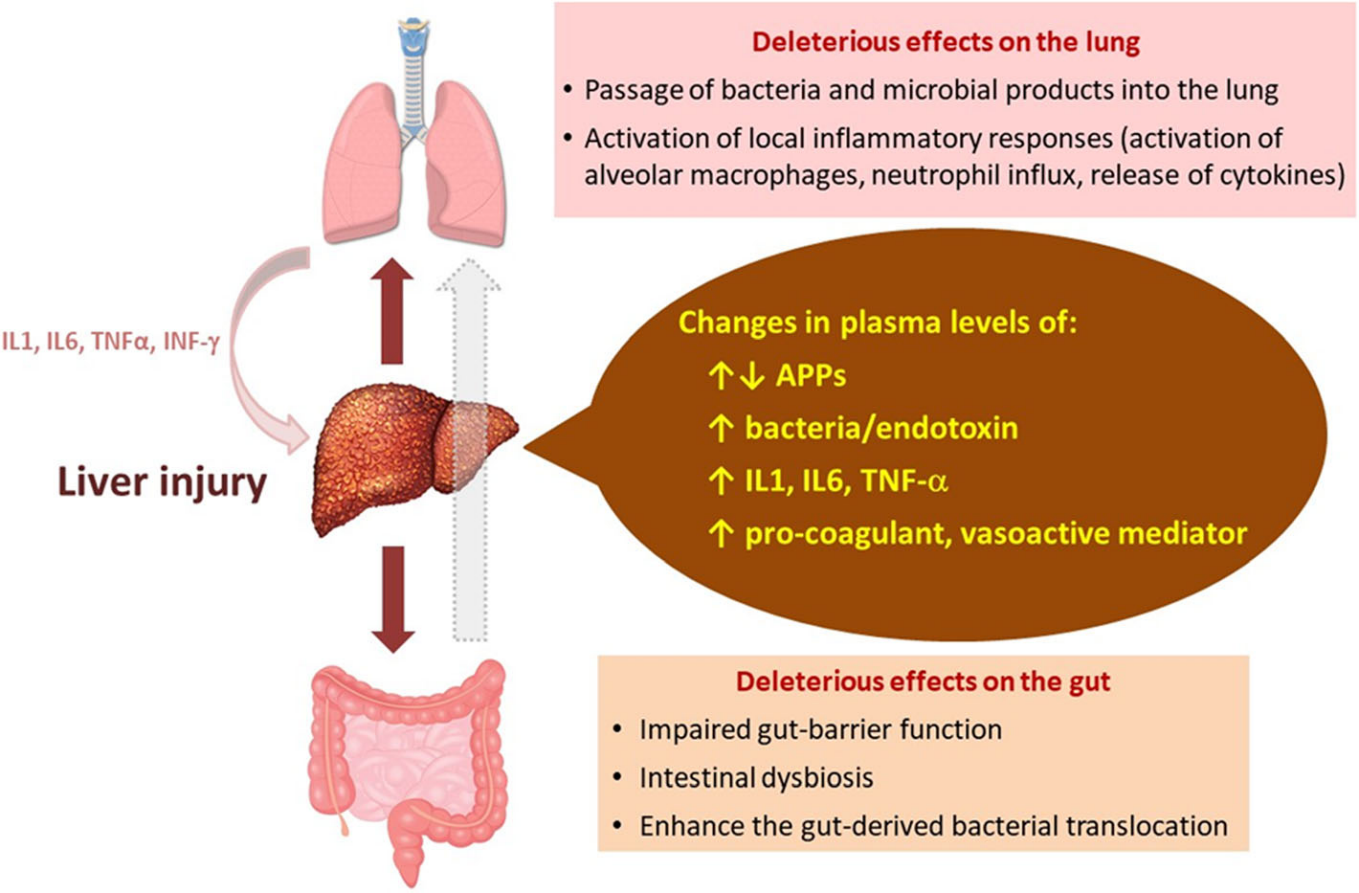
Liver damage contributes to the development of acute respiratory distress syndrome. Liver injury leads to changes in the expression of acute-phase proteins (APPs) and to an increase in plasma levels of bacteria/bacterial products, pro-inflammatory cytokines, and pro-coagulant and vasoactive factors in the lung and systemic circulation. These mediators generate deleterious effects on the lung (passage of bacteria /bacterial products and inflammation) and on the gut (intestinal dysbiosis, impairment of gut barrier integrity, leakage of bacteria/bacterial products into the portal circulation and into the mesenteric lymph), resulting in relevant changes in the hepatic and pulmonary microbiota and promoting inflammation and oxidative stress in liver and lung tissues. In addition, lung-derived cytokines promote the synthesis of APPs and activation of inflammatory responses in the liver. All these responses mediated by the gut–liver–lung axis contribute to lung injury and multiple organ dysfunction in critical illness. *IL* interleukin, *TNF* tumor necrosis factor, *INF* interferon

Pulmonary deposition of intravascular bacteria, and their products alter the structure and function of the lung by different mechanisms including (i) direct cytotoxic effect on alveolar epithelial and endothelial cells, (ii) modulation of local innate immune responses in the lung via activation of toll-like receptors (TLRs), resulting in activation of resident alveolar macrophages and neutrophil influx and in the production of reactive oxygen species, (iii) activation of the coagulation cascades and platelet aggregation, leading to pulmonary microvascular thrombosis [29, 48, 49, 55–59], and (iv) a sustainable increase in pulmonary vascular resistance [22, 30] (Fig. 2). All these mechanisms alter the alveolar endothelial and epithelial cell functions and enhance barrier permeability leading to the formation pulmonary alveolar edema and respiratory failure [14, 60], the two main characteristics of ARDS (Fig. 2).

### Metabolic inactivation and detoxification of endogenous inflammatory mediators

The hepatobiliary system has an important capacity to inactivate and detoxicate pro-inflammatory cytokines, vasoactive mediators, and eicosanoids from the systemic circulation. Removal of all these mediators constitutes a critical element of systemic and pulmonary host defense, protecting the lung and other extrahepatic organs from injury (Fig. 1) [8–10, 46]. Like endotoxin, increased levels of cytokines (such as IL-8, IL-1β, ENA-78, TNF-α, MCP-1, MIP-1α,…) and arachidonic acid-derived eicosanoids (thromboxane, leukotrienes) not cleared by the liver have been shown to exert a direct cytotoxic effect on alveolar epithelial and endothelial cells, to activate local innate immune responses and to promote platelet aggregation in the lung, contributing to the development of diffuse alveolar damage (DAD) [55–59].

### Hepatic synthesis of inflammatory mediators that can activate pulmonary alveolar macrophages and, consequently, increase inflammation in the lung

Hepatic mononuclear cells include a heterogeneous population of lymphocytes, Kupffer cells (hepatic resident macrophages), monocytes, and granulocytes that perform vital functions for the innate and adaptive immune system. In response to injury, activation of these hepatic mononuclear cells enhances the production and release of inflammatory mediators, such as IL-1, IL-6, TNF-α, platelet-activating factor (PAF), and leukotrienes, into the systemic circulation [61], where they play an important role in the lung–liver interaction [18, 31, 51, 61–64]. These liver-derived inflammatory mediators alter lung structure function early in acute inflammatory diseases (such as sepsis) and contribute to some extent to lung damage upon activation of pulmonary alveolar macrophages (Fig. 1] [17, 65]. In this line, elevated levels of TNF-α and IL-1β, two cytokines that are mainly synthetized by alveolar macrophages, have been found in the lungs of rats with carbon tetrachloride (CCl4)-induced cirrhosis, along with an increase in lipid peroxidation (TBARS) and antioxidant enzymes (superoxide dismutase and catalase) in the liver and lung tissues. These events are also associated with altered gas exchange and changes in the size of pulmonary vessels in these rats [66, 67]. Besides high levels of endotoxin [50, 68], patients with liver disorders also have high circulating levels of TNF-α, IL-1, and IL-6 [51–54] because of the altered capacity for inactivation and detoxification and the increased synthesis of pro-inflammatory mediators by the liver [9, 44, 46, 61]. These specific cytokines have been shown to modulate systemic inflammatory responses and participate in the development of lung damage [69–72]. Therefore, it is possible that cytokines of hepatic origin may control and modulate the local host defense and immune system of the lung, contributing to lung injury (Figure 2).

### The liver is the main organ responsible for the acute-phase response

The organism responds to tissue injury or infection by local changes such as those associated to inflammation and by a coordinated sequence of systemic and metabolic process, known as the acute-phase response, aimed to restore homeostasis and recover from injury [63, 73–76]. One of the mayor characteristics of this acute-phase response is a change in plasma concentration (either increase or decrease) of the acute-phase proteins (APPs) expressed in the liver [74]. Cytokine-driving synthesis of acute-phase proteins in the liver modulates the systemic and pulmonary host inflammatory responses and intermediary metabolism (Fig. 1) [22, 30, 48]. The hepatic APPs have a variety of functions that include microbicidal and phagocytic activity (e.g., LPS binding protein, complement components, C-reactive protein), recruitment of immune cells to inflammatory sites (e.g., serum amyloid A), hemostasis (e.g., fibrinogen, α1-acid glycoprotein), antioxidant, and prevention of iron loss (e.g., haptoglobin) and anti-proteolytic actions to counterbalance protease activity at sites of inflammation (e.g., α2-macroglobulin, α1-antitrypsin, and α1-antichymotrypsin) (Fig. 1) [63, 74, 75].

While the local inflammation occurs in the alveolar airspaces of patients with ARDS, the acute-phase response is induced in the liver [73, 76, 77]. Interestingly, in pneumonia-induced ARDS, this liver-derived acute-phase response occurs independently of bacterial dissemination and depends instead on inflammatory signaling molecules derived from the pulmonary immune cells, such as the cytokines IL-1, IL-6, and TNF-α [10, 62, 63, 73, 74]. Then, these lung-derived cytokines can travel from the lung into the systemic circulation and ultimately modify acute-phase gene expression in the liver [63, 73, 78, 79] upon activation of the transcription factors STAT3 (signal transducer and activator of transcription 3) by IL-6 and activation of RelA (v-rel avian reticuloendotheliosis viral oncogene homolog A, also known as NF-kB3) by the early-response cytokines TNF-α and IL-1 [73, 78, 79]. In response to these cytokines, the liver changes the expression of many acute-phase proteins such as C-reactive protein, α-1 antitrypsin, serum amyloid A protein, and others [10, 62, 63, 74, 77], which in turn can directly travel back to the lung and pass into the airspaces where they cause inflammation, predominantly via activation of alveolar macrophages (Fig. 2) [5, 14]. These phagocytic cells are targeted by multiple hepatic APPs such as SAA [80, 81], SAP [82], LBP [83], and C-reactive protein [84, 85]. Once activated by these hepatic APPs, alveolar macrophages release cytokines (IL-6 and CXCL1) that enhance local inflammation, in part by promoting neutrophil influx to the insterstitium and alveolar airspaces. Excessive inflammation in the alveoli may result in an increase in oxidative stress and lung injury [86, 87]. Besides this potentially deleterious effect, hepatic APPs at the site of plasma extravasation has other potential functions, including opsonization of bacteria, leukocyte activation, antiprotease, antioxidant activities, and modulation of the coagulation pathway [63, 75]. These mechanisms help to regulate host defense, limit excessive inflammation and immune responses, and promote bacterial clearance, preventing infection dissemination and reducing the risk of organ damage in the setting of pneumonia and sepsis. Also, hepatic APPs exert liver protection by countering TNF-dependent toxicity in the liver and attenuate systemic inflammation and mortality in sepsis and pneumonia-induced ARDS (Fig. 1) [79, 86, 88–90]. Altogether, the bidirectional liver–lung axis mediated by APPs is critical for integrating systemic and pulmonary responses, balancing regulation of multiple host defenses and activation of inflammation to restore homeostasis and recover from organ injury [48, 61, 86]. Disbalance in this liver–lung communication can be an important factor in the initiation and progression of ARDS and of the damage to other organs [73].

### Nutrients, bile, and hormone production

The liver plays an important role in regulating metabolic homeostasis and in the synthesis and processing of lipids and carbohydrates that supply energy to other organs [91]. It is also the major site of synthesis of key proteins and bile acids that are critical for the normal uptake of vitamins and lipids [92]. Therefore, alterations in the flux of carbohydrates and lipids through the liver can indirectly impact distal organs due to alteration of their energy statuses [93]. In addition, hyperbilirubinemia in the context of liver diseases has been shown to cause some lung-specific deleterious effects, by entering the lung tissue, reaching the alveolar airspaces, and deteriorating the surface tension properties of the alveolar surfactant [94]. Although bilirubin has antioxidant properties, high bilirubin levels can also activate oxidative stress, apoptosis, and inflammatory responses in different cell types and organs [95–98]. Therefore, hyperbilirubinemia may actively participate in the development of ARDS, although the underlying mechanisms have not been fully elucidated. Finally, the liver produces several hormones that mediate diverse extrahepatic effects, such as insulin-like growth factor, angiotensinogen, and thrombopoietin, which have been shown to influence the development of ARDS (Fig. 1) [55, 99, 100].

### The gut–liver–lung axis

The pathogenic mechanisms of ARDS should be considered within a gut–liver–lung axis. Growing evidence indicates that intestinal microbiota and the mucosal immune system of the gut have an important impact on the function of the gastrointestinal tract itself and extra-intestinal organs, such as the lung and the liver [29, 48, 49]. Liver cirrhosis and other liver diseases favor the gut-derived bacterial translocation into the liver and lung by several mechanisms (Fig. 2).

First, patients with liver disorders have intestinal dysbiosis characterized by a significant shift of the microbial composition toward pro-inflammatory bacteria. This gut dysbiosis is accompanied by activation of local intestine immune responses and impaired gut barrier function. A leaky gut barrier facilities bacterial translocation of live bacteria or their microbial products from the intestinal lumen to the liver, via portal circulation, and to systemic circulation and the lung via the mesenteric lymphatic system [29, 101]. In the lung and the liver, gut microbiota can directly modulate their local immune cells (mainly alveolar macrophages and Kupffer cells, respectively) via activation of toll-like receptors (TLRs) and indirectly via different bacterial metabolites and signaling molecules, such as PAMPs [29, 48, 49, 90]. Activated alveolar macrophages in the lung and Kupffer cells in the liver release pro-inflammatory cytokines, contributing to the initiation and/or progression of lung and liver damage and activation of systemic inflammation [29, 101], which can also cause dysfunction in other organs.

Second, liver dysfunction can imply less capacity of the liver to remove bacteria, bacterial products, and inflammatory mediators from circulation, leading to increased levels of these molecules in blood.

Third, this pathological gut-derived bacterial translocation could cause important changes in the lung microbiome (Fig. 2) [90, 102, 103]. Indeed, pulmonary microbiome is frequently enriched with gut-related bacteria (*Bacteroidetes* and *Enterobacteriaceae*) in critically ill patients [102, 103]. As a consequence of liver diseases, this gut-derived bacteria and accumulation of PAMPs, cytokines and other pro-inflammatory molecules in the systemic circulation can potentially cause or exacerbate lung injury upon TLR-4-mediated activation of intravascular and alveolar macrophages within the lung and recruitment of neutrophils and direct toxic effects of bacterial products on pulmonary microvasculature (Fig. 2) [68, 102–108]. Altogether, the gut–liver–lung axis seems to exert a relevant role in the initiation and modulation of hepatic, pulmonary, and systemic immune responses that contribute to the damage of the liver, the lung, and other organs.

### Extracellular vesicles

Extracellular vesicles (EVs), a term that includes microvesicles (MVs), exosomes, and apoptotic bodies, represent an emerging mechanism of interorgan communication in many diseases, including liver diseases and ARDS [106, 109–111]. Extracellular vesicles are defined as membrane-bound vesicles, ranging 0.1–1.0 μm in diameter, which are released from cells by the budding of the cellular plasma membrane and carrying a diverse cargo, including lipids, proteins, RNAs, and miRNAs. The EVs are recognized as important mediators of interaction between different organs, and they are considered attractive therapeutic targets in different diseases [112, 113]. Notably, the levels of circulating EVs have been reported to be increased both in patients with cirrhosis and ARDS. However, the potential role of circulating EVs in mediating liver–lung communication in the context of ARDS is not currently understood, representing an interesting topic for further investigation.

## Conclusions

Liver injury and hepatotoxicity occur frequently in critically ill patients, and significantly influence their prognosis. Patients with severe hepatic dysfunction are at high risk for irreversible ARDS because of multiple defects in host defense and dysregulation of inflammatory responses. Interrelations between hepatic and pulmonary functions influence the development and progression of ARDS and play a central role in the resolution of lung damage by several mechanisms. First, the liver regulates host defense and modulates systemic inflammation. Also, the liver activates acute inflammatory responses in the lung early in the development of ARDS. Although promoting inflammation can be detrimental in the context of acute lung injury, the liver response to an inflammatory insult is also pro-defense and pro-survival. The understanding of the complex relation between the liver and the lung requires further research in order to improve the clinical management and to identify new diagnostic and therapeutic options for patients with or at risk for ARDS.

## Data Availability

Not applicable

## Abbreviations

ARDS: Acute respiratory distress syndrome
DAD: Diffuse alveolar damage
ICU: Intensive care unit
AST: Aspartate aminotransferase
ALT: Alanine aminotransferase
ALP: Alkaline phosphatase
INR: International normalized ratio
PAMPs: Pathogen-associated molecular patterns
TLRs: Toll-like receptors
IL: Interleukin
ENA-78: Epithelial neutrophil-activating peptide-78
TNF: Tumor necrosis factor
MCP-1: Monocyte chemoattractant protein-1
MIP-1α: Macrophage inflammatory protein-1α
PAF: Platelet-activating factor
CCl4: Carbon tetrachloride
TBARS: Thiobarbituric acid-reactive substances
APPs: Acute-phase proteins
LPS: Lipopolysaccharides
STAT3: Signal transducer and activator of transcription 3
RelA: v-rel avian reticuloendotheliosis viral oncogene homolog A
NF-kB3: Nuclear factor kappa-light-chain-enhancer of activated B3
SAA: Serum amyloid A
SAP: Serum amyloid P
LBP: Lipopolysaccharide binding protein
CXCL1: Chemokine (C-X-C motif) ligand 1
EVs: Extracellular vesicles
MVs: Microvesicles
RNAs: Ribonucleic acids
miRNAs: Micro-ribonucleic acids
